# Deep learning methods for protein torsion angle prediction

**DOI:** 10.1186/s12859-017-1834-2

**Published:** 2017-09-18

**Authors:** Haiou Li, Jie Hou, Badri Adhikari, Qiang Lyu, Jianlin Cheng

**Affiliations:** 1 Department of Computer Science and Technology, Soochow University, Suzhou, Jiangsu 215006 China; 2 Department of Electrical Engineering and Computer Science, University of Missouri, Columbia, MO 65211 USA; 3 Department of Mathematics and Computer Science, University of Missouri–St. Louis, 1 University Blvd. 311 Express Scripts Hall, St. Louis, MO 63121 USA

**Keywords:** Deep learning, Deep recurrent neural network, Restricted Boltzmann machine, Protein torsion angle prediction

## Abstract

**Background:**

Deep learning is one of the most powerful machine learning methods that has achieved the state-of-the-art performance in many domains. Since deep learning was introduced to the field of bioinformatics in 2012, it has achieved success in a number of areas such as protein residue-residue contact prediction, secondary structure prediction, and fold recognition. In this work, we developed deep learning methods to improve the prediction of torsion (dihedral) angles of proteins.

**Results:**

We design four different deep learning architectures to predict protein torsion angles. The architectures including deep neural network (DNN) and deep restricted Boltzmann machine (DRBN), deep recurrent neural network (DRNN) and deep recurrent restricted Boltzmann machine (DReRBM) since the protein torsion angle prediction is a sequence related problem. In addition to existing protein features, two new features (predicted residue contact number and the error distribution of torsion angles extracted from sequence fragments) are used as input to each of the four deep learning architectures to predict phi and psi angles of protein backbone. The mean absolute error (MAE) of phi and psi angles predicted by DRNN, DReRBM, DRBM and DNN is about 20–21° and 29–30° on an independent dataset. The MAE of phi angle is comparable to the existing methods, but the MAE of psi angle is 29°, 2° lower than the existing methods. On the latest CASP12 targets, our methods also achieved the performance better than or comparable to a state-of-the art method.

**Conclusions:**

Our experiment demonstrates that deep learning is a valuable method for predicting protein torsion angles. The deep recurrent network architecture performs slightly better than deep feed-forward architecture, and the predicted residue contact number and the error distribution of torsion angles extracted from sequence fragments are useful features for improving prediction accuracy.

**Electronic supplementary material:**

The online version of this article (10.1186/s12859-017-1834-2) contains supplementary material, which is available to authorized users.

## Background

The conformation of the backbone of a protein can be largely represented by two torsion angles (phi and psi angles) associated with each Cα atom. A number of methods, mostly data-driven machine learning methods, have been developed to predict torsion angles from protein sequences; and the predictions can then be used as restraints to predict protein tertiary structures.

The first real-value psi angle prediction method based on machine learning, DESTRUCT, was proposed by Wood and Hirst [1] in 2005. It used protein sequence profile - position specific scoring matrices (PSSM) - as input with iterative neural networks to predict psi angle. Real-SPINE2.0 was the first method to predict both phi and psi angles using neural network [2]. ANGLOR used neural networks to predict phi angle and support vector machines to predict psi angle separately [3].

Some recent methods enhanced or integrated standard machine learning methods such as neural networks and support vector machines (SVM) to improve torsion angle prediction. Real-SPINE3.0 used a guided-learning mechanism to training a two-layer neural network to reduce Mean Absolute Error to 22° for phi angle and 36° for psi angle [4]. TANGLE used a two-level SVM based regression approach to make prediction [5]. SPINE X [6] and SPINE XI [7] combined discrete and continuous real-value prediction of torsion angle with multi-step neural network learning, which yielded a MAE of 35° and 33.4° for phi and psi angles, respectively. A comprehensive study has shown that SPINE X performs better than ANGLOR and TANGLE, especially on psi angle prediction [8].

In recent years, deep learning methods that overcome some limitations of traditional artificial neural networks have been successfully applied to predict local and non-local structural properties of proteins [9–12]. SPIDER2 that used an iterative deep learning method further reduced the MAE of phi and psi angle prediction [13]. However, most existing methods for torsion angle prediction are restricted to learning structural properties from local residue information in sliding windows. A recent method tried to explore the long-range non-local interaction among residues by utilizing bidirectional neural networks [14], which has shown that non-local contact information of residues can significantly improve the torsion angle predictions.

In this study, we developed four deep learning methods, including Deep Neural Network, Deep Recurrent Neural Network, Deep Restricted Boltzmann Machines, and Deep Recurrent Restricted Boltzmann Machines to predict torsion angles. To improve the prediction accuracy, various combinations of different input features including two novel features (predicted contact number and error distribution of torsion angles of sequence fragments) are systematically examined and compared. We also compared our methods with two other torsion angle predictors: SPIDER2 [13] and TANGLE [5]. Our main contributions of this work include: (1) introducing two novel features that are useful for protein torsion angle prediction; and (2) developing and evaluating different deep learning architectures for torsion angle prediction.

## Methods

### Datasets

In order to objectively compare our four methods and previous methods, we created a new dataset that has no overlap with the datasets used to training the existing methods in the literature. We obtained protein sequences released between June 2015 and March 2016 from Protein Data Bank (PDB) [15] and removed sequences whose length is out of range [30,500]. We then removed redundant sequences to make sure the pairwise sequence identity between two sequences is less than 25%, resulting in a dataset consisting of 1652 protein sequences. We randomly selected 100 proteins from this dataset to estimate the distribution of errors between the torsion angles predicted from sequence fragments generated by FRAGSION [18] and true torsion angles for each of 20 residue types (see section Input features for details). From the remaining 1552 protein sequences, we randomly chose 232 sequences as test dataset, and the rest as training dataset. The training dataset has 1320 sequences.

In order to further assess the performance of the different methods, we selected 11 free-modeling targets in the most recent CASP12 as an independent test dataset, whose native structure are available for torsion angle evaluation (see Additional file 1: Table S1 for the list of CASP12 targets and their length).

### Input features

**Fig. 1:**
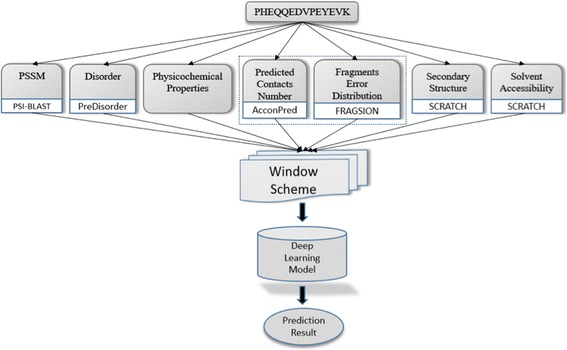
The flowchart of the deep learning methods for protein torsion angle prediction. Five commonly used features and two new features are used as input to build our deep learning method to predict torsion angles

Figure 1 illustrates the general flowchart of our deep learning approach for torsion angle prediction. In our methods, seven different types of features represent each residue in a protein. The seven features include: physicochemical properties, protein position specific scoring matrix, solvent accessibility, protein secondary structure, protein disorder, contact number, and the error distribution of torsion angles predicted from sequence fragments. The first five features have been commonly used in various protein prediction problems such as secondary structure prediction and torsion angle prediction before. But the last two features are two novel features used for torsion angle prediction for the first time. The details of these features are described as follows.

**Fig. 2:**
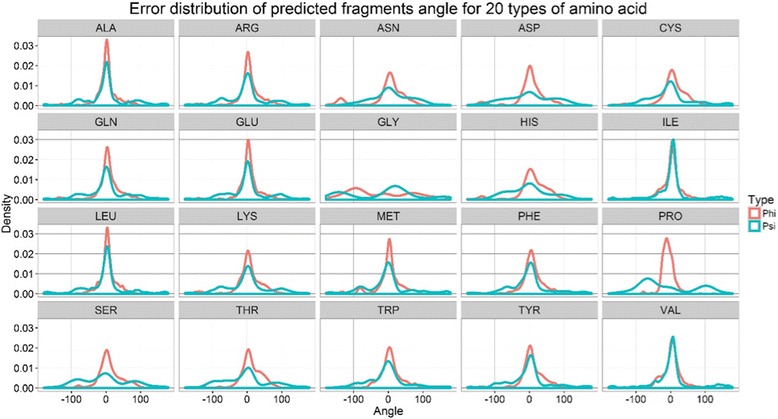
The error distributions of torsion angles predicted from fragments for 20 types of amino acids. The red and green lines represent the distribution of phi and psi angles, respectively. The x-axis represents angles in the range [−180,180], and y-axis is the density

1. Physicochemical properties: 7 numerical values representing sequence-independent physicochemical properties of each residue type [16, 17], including steric parameter, polarizability, normalized van der Waals volume, hydrophobicity, isoelectric point, helix probability and sheet probability.
2. Protein position specific scoring matrix (PSSM): a sequence profile generated from the multiple sequence alignment between a target sequence and its homologous sequences found by PSI-BLAST [18], which is commonly used for various protein prediction problems such as protein secondary structure prediction, solvent accessibility prediction, and residue contact prediction.
3. Solvent accessibility: the solvent accessibility state (buried or exposed) of each residue [19], which was predicted by SCRATCH [20].
4. Secondary structure: the secondary structure state of each residue, which was predicted by SCRATCH [20]. SCRATCH can predict both 3-state secondary structure and 8-state secondary structure.
5. Protein disorder: the disorder probability of each residue, which was predicted by PreDisorder [21].
6. Contact number: the number of residues that each residue may be in contact with. Contact number is an important constraint related to protein folding. It has been suggested that, given the contact number for each residue, the number of protein conformations that satisfy the contacts number constraints are very limited [22]. Thus, the contact numbers of a protein may serve as useful restraints for de novo protein structure prediction [23]. We hypothesize that contact numbers are useful for protein torsion angle prediction. We used AcconPred to predict contacts numbers for each residue [23]. The prediction can be either a real-value contact number or probabilities of 15 contact number labels.
7. The estimated probability density function of errors (difference) between true torsion angles and predicted torsion angles based on related sequence fragments. The statistics was estimated from 100 randomly selected proteins. A sequence fragment is a short segment of consecutive residues in a protein, typically 3 to 15 residues long. Because the structures of similar sequence fragments are often similar, sequence fragments have been widely used in protein homology modeling [24], de novo structure prediction [25], and structure determination [25]. Some tools have been developed for generating sequence fragments for any protein, such as Rosetta fragments generator [26] and FRAGSION [27]. Here, we used FRAGSION to generate 200 3-mer fragments for each position of a protein, and calculate the mean value of phi and psi angles from the angles of the 200 fragments as predicted phi and psi angles for each residue. We use the estimated probability density function of errors (difference) between predicted and true torsion angles of 100 selected proteins in a dataset as a feature. The randomly chose 100 sequences in the dataset have less than 25% identity with the training and test datasets. We calculated the errors between the angles predicted from sequence fragments (*P*) and true torsion angles (*T*) of all residues of these proteins. The probability density function of errors was generated for each residue type. The distributions of errors for all 20 types of residues are shown in Fig. 2. According to the figure, the errors of predicted phi angles and psi angles of some residue types like GLN, ALA and PRO largely follow the normal distribution, while the error distribution of predicted angles of other residues such as GLY and HIS is not normal. For those residues whose errors follows the normal distribution, we used equation: $$ \left\{\begin{array}{c}\alpha =P- avg\\ {}\beta = std\kern2.5em \end{array}\right. $$ to generate two raw features, where *P* represents angle predicted from fragments, *avg.* the average error and *std.* the standard deviation of the error. For other residues like residues CYS, ASP, GLY, HIS, ASN, SER and THR, we let their *avg.* equal to 0 and standard deviation equal to 0, and use the same equation to generate two raw features. Finally, we convert the α and β of phi and psi angles into normalized features using both sin and cos function, which are used as input to deep learning.

### Encoding scheme

The input features were normalized to the range of 0 to 1. The experimental values of phi and psi torsion angles of each protein were calculated by the DSSP program [28], which are the target output. There are four output nodes to predict the sine and cosine of the phi and psi angles, i.e. sin(*φ*) , cos(*φ*) , sin(*ψ*) , cos(*ψ*), respectively. Sine and cosine were employed to remove the effect of angle periodicity during training. Predicted sine and cosine values of the two angles can be readily converted back to angles by using the equation: *φ* = *tan* ^−1^[sin(*φ*)/ cos(*φ*)].

Since its nearby residues could influence the torsion angle of a residue, a sliding window approach was used to extract the features for each residue. We combine all of the features in the window to form a feature vector for any residue *i*. For example, if window size *w* is 15, letting *k* = (*w*-1)/2 = 7, we combine the features: *F(i-k), F (i-k + 1), ..., F(i), ..., F(i + k-1), F(i + k)* into one feature vector for residue *i*. In the past, the selection of a suitable window size was largely carried out in a heuristic way. ANGLOR [13] chose a window size of 21 residues, and SPIDER2 [12] chose a window size of 17 residues, while TANGLE [14] used a window size of 9 and 13 residues for phi and psi separately. In our experiments, we examined the performance of different window sizes ranging from 3 to 17, and then chose an optimal window size for each method based on 5-fold cross validation on the training data.

### Deep learning methods

**Fig. 3:**
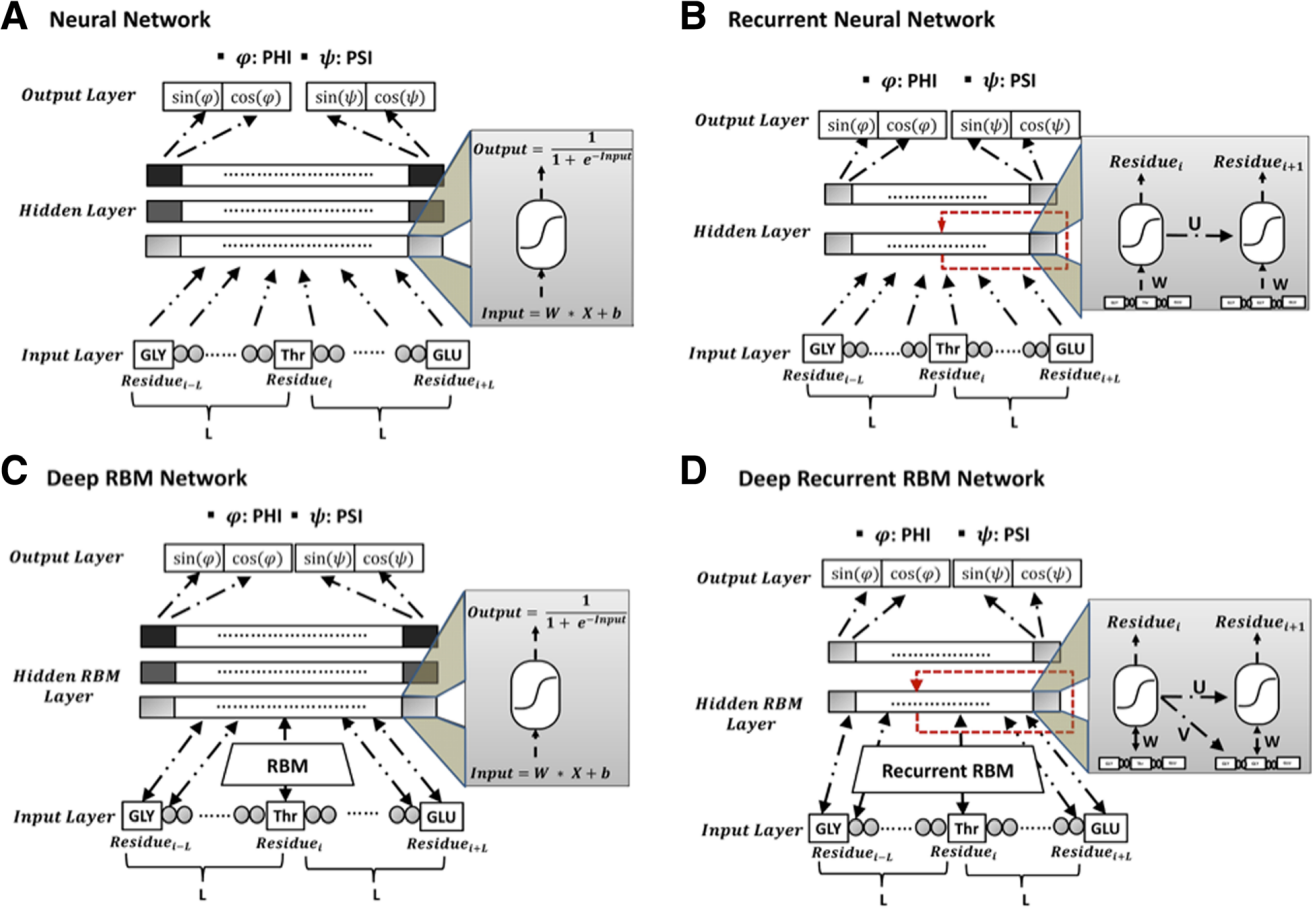
The architectures of four deep learning networks for torsion angle prediction. **a** Deep neural network (DNN). Features in the input layers are mapped to the hidden layers by sigmoid functions, from hidden layer to hidden layer, and finally propagated to the output layer for prediction. The weights in the network are randomly initialized according to the uniform distribution. The architecture is fine-tuned by back-propagation with SFO algorithm. **b** Deep recurrent neural network (DRNN). The inputs are connected to the first hidden layer by weight matrix W, and the neighboring positions in the first hidden layer are inter-connected by weight matrix U. The parameters are randomly initialized. The architecture is optimized by back-propagation through time with SFO algorithm. **c** Deep restricted Boltzmann (belief) network (DRBM). The layers are stacked with each other through Restricted Boltzmann Machine. And the weights are pre-trained by RBM. Predictions are made by forward pass and the network is optimized by back-propagation with SFO algorithm. **d** Deep Recurrent RBM network. Forward-propagation and backward-propagation follows the same strategy as deep recurrent neural network, while the parameters are pre-trained by RBM

Deep learning, a new set of machine learning algorithms closely related to artificial neural networks [4, 9, 10, 29–33], has achieved the state-of-the-art performance in many problems, and is getting more and more popular in bioinformatics [9–12]. Here, we designed 4 types of deep learning architectures for torsion angle prediction. Our deep learning architectures include deep feed-forward neural network, deep recurrent neural network, deep belief network in which the parameters are pre-trained by restricted Boltzmann machine and deep recurrent RBM network where the RBM is trained to initialize the parameters in recurrent neural network. The four deep learning architectures are visualized in Fig. 3. The network consists of an input layer, hidden layers and an output layer. Arrows represent connections between layers. In the input layer, the nodes (neurons) represent the features of each residue in a sequence window centered on a target residue for which torsion angles are predicted. All inputs are connected to every node in the adjacent hidden layer. The nodes in a hidden layer are fully connected to the nodes in next layer, and finally the nodes in the last hidden layer are fully connected to the four output nodes, corresponding to sin(*φ*) , cos(*φ*) , sin(*ψ*) , cos(*ψ*). The nodes in the hidden layers are activated by the sigmoidal function. The four different deep learning architectures used in this study are described in details below.

#### Deep neural network

The deep neural network is a standard multi-layer feed-forward network, consisting of one visible “input” layer, multiple intermediate “hidden” layers, and one predicted “output” layer [34], as visualized in Fig. 3a. The nodes in each layer are fully connected to the nodes in its adjacent layers, and the network propagates the inputs from first visible layer to the last output layer in a forward manner. For each node in the hidden layers, the input is calculated as the values of nodes in the previous layer multiplied by weight matrix, which is the weighted sum of the previous layer and is adjusted by the bias offset. The non-linear sigmoid activation function is applied to calculate the output of a node from its input, which is defined as:

1 $$ \left\{\begin{array}{c}\kern0.5em Input\kern0.75em Layer:\kern2.00em {O}_i^{(0)}={Input}_i\kern9em \\ {}\  Hidden Layer:\left\{\begin{array}{c}{I}_i^{(n)}={\sum}_j{W_{ij}}^{\ast }{O}_j^{\left(n-1\right)}+{b}_i^{(n)}\kern0.5em \\ {}{O}_i^{(n)}=\frac{1}{1+{e}^{-{I}_i^{(n)}}}\kern7.25em \\ {}i,j=1,2,\dots .k;\kern0.5em n=2,\dots .,N-1\end{array}\right.\\ {} Output Layer:\kern1.25em {O}_i^{(N)}={\sum}_j{W_{ij}}^{\ast }{O}_j^{\left(N-1\right)}+{b}_i^{(N)}\kern0.5em \end{array}\right. $$

where the ${I}_i^{(n)}$, ${O}_i^{(n)}$ and ${b}_i^{(n)}$ are the input, output and bias of *i* ^*th*^ node in the *n* ^*th*^ layer, respectively, and (*W*, *b*) is the weight and bias. The network sums up all the non-linear outputs of nodes from one layer and propagates to next hidden layer until reaching final output layer. The linear function is applied to the nodes in the output layer to generate predicted real-value torsion angles.

The forward pass in the neural network generally is followed with the backward pass that propagates the errors between the true and predicted torsion angles back to lower-level layers through the network, and updates the weights and biases according to the partial derivative of the error with respect to them (i.e. gradient) to minimize the prediction error (or energy) [35]. In this study, the energy (loss) function is defined as the least square error between predicted angles and true angles:

2 $$ E\left(W,b\right)=\frac{1}{2}\sum {\left({O}_{prediction}-{O}_{true}\right)}^2\kern21em $$

The gradients of all weights and biases are inferred from back-propagation. That is, given a network with one hidden layer, the parameters in the network can be updated as:

3 $$ \left\{\begin{array}{c}\frac{\partial E}{\partial {W}_{ij}^{(2)}}={S_i}^{\ast}\left({O}_j^{prediction}-{O}_j^{true}\right)\kern12.75em \\ {}\frac{\partial E}{\partial {b}_j^{(2)}}=\left({O}_j^{prediction}-{O}_j^{true}\right)\kern14.75em \\ {}\frac{\partial E}{\partial {W}_{ij}^{(1)}}={X_i}^{\ast }{S_j}^{\ast}\left(1-{S}_j\right){\sum}_{k=1}^K\partial {W}_{jk}^{(2)}\left({O}_k^{prediction}-{O}_k^{true}\right)\\ {}\frac{\partial E}{\partial {b}_j^{(1)}}={S_j}^{\ast}\left(1-{S}_j\right){\sum}_{k=1}^K\partial {W}_{jk}^{(2)}\left({O}_k^{prediction}-{O}_k^{true}\right)\kern2.75em \end{array}\right.\kern8.75em $$

Where ${W}_{ij}^{(1)}$and ${b}_j^{(1)}$ are the weight and bias in the first layer, ${W}_{ij}^{(1)}$ connecting node *i* in input layer to node *j* in hidden layer, and ${W}_{ij}^{(2)}$and ${b}_j^{(2)}$ are the weight and bias in the second layer. *S*_*i*_ is the data of node *i* in the hidden layer, and ${O}_j^{prediction},{O}_j^{true}$are the predicted and true torsion angles. All the parameters are updated by Sum-of-Functions-Optimizer (SFO) optimization method [36].

#### Deep restricted Boltzmann machines

Traditional neural network starts by randomly initializing the weights of networks, which are optimized by the back-propagation over all data. Training deep neural networks in this way suffers the problem of gradient vanishing or exploding during back-propagation in deep networks, and slow convergence with randomly initialized weights to poor local optima [37]. Unsupervised pre-training methods have been developed to address this limitation, such as pre-training with denoising auto-encoders [38], or pre-training with restricted Boltzmann machines (RBMs) [39], which provide good initialization of parameters in network that speed up and enhance training of very deep networks. In this study, we applied our in-house Deep Belief Network toolbox [12], a deep network with stacked restricted Boltzmann machines (RBM), to torsion angle prediction problem, as visualized in Fig. 3c.

A RBM is a generative model that can model the probability distribution over the binary data or real-valued continuous data [39–41]. A RBM is a two-layer network, consisting of one visible layer and one hidden layer, which represents the distribution of input data over all possible hidden units *P*(*v*) =  ∑ *P*_*h* ∈ *H*_(*v*, *h*). The objective of training a RBM is to adjust the weights of RBM in order to maximize the likelihood of the data - *P*(*v*). The training of RBM is completely energy-guided based on the joint probability of all visible and hidden nodes, which is described by the following equation:

4 $$ \left\{\begin{array}{c}E\left(v,h\right)=-\sum \limits_i{b}_i{v}_i-\sum \limits_j{c}_j{h}_j-\sum \limits_{i,j}{h}_j{v}_j{w}_{ij}\\ {}p\left(v,h\right)=\frac{e^{-E\left(v,h\right)}}{\sum \limits_{h,v}{e}^{-E\left(v,h\right)}}\\ {}p(v)=\sum \limits_h\frac{e^{-E\left(v,h\right)}}{\sum \limits_{h,v}{e}^{-E\left(v,h\right)}}\\ {}p(h)=\sum \limits_v\frac{e^{-E\left(v,h\right)}}{\sum \limits_{h,v}{e}^{-E\left(v,h\right)}}\end{array}\right. $$

where the *v*_*i*_ and *h*_*j*_ denote the value of *i* ^*th*^ visible node and *j* ^*th*^ hidden node, *b*_*i*_ and *c*_*j*_ are the bias of *i* ^*th*^ visible unit and *j* ^*th*^ hidden node, and *w*_*ij*_ is the weight connecting the *i* ^*th*^ visible node and *j* ^*th*^ hidden node.

To train the RBM, the parameters <*W,b,c* > are updated by the gradient of the negative log-likelihood of the data with respect to the parameters, which is given by:

5 $$ \left\{\begin{array}{c}\frac{\partial }{\partial \theta}\left(- lnP(v)\right)=\sum \limits_h\left[P{\left(h|v\right)}^{\ast}\left(\frac{\partial E\left(v,h\right)}{\partial \theta}\right)\right]-\sum \limits_{h,v}\left[P{\left(h|v\right)}^{\ast}\left(\frac{\partial E\left(v,h\right)}{\partial \theta}\right)\right]\\ {}P\left(h|v\right)=\frac{e^{-E\left(v,h\right)}}{\sum \limits_h{e}^{-E\left(v,h\right)}}\ \\ {}P\left(v|h\right)=\frac{e^{-E\left(v,h\right)}}{\sum \limits_v{e}^{-E\left(v,h\right)}}\end{array}\right. $$

The gradient of each parameters were further approximated and calculated by the contrastive divergence (CD) algorithm [42], which has shown fast convergence within few iterations of Gibbs sampling. In our experiment, the parameters are updated as:

6 $$ \left\{\begin{array}{c}\frac{\partial lnP\left(\theta \right)}{\partial {W}_{ij}}=P\left({h}_j=1\ \right|{v}^{(0)}\left){}^{\ast }{v}_i^{(0)}-\frac{1}{k}{\sum}_{k=1}^KP\left({h}_j=1\ \right|{v}^{(k)\ast}\right){v}_i^{(k)}\ \\ {}\frac{\partial lnP\left(\theta \right)}{\partial {b}_i}={v}_i^{(0)}-\frac{1}{k}{\sum}_{k=1}^K{v}_i^{(k)}\\ {}\frac{\partial lnP\left(\theta \right)}{\partial {c}_j}=P\left({h}_j=1\ \right|{v}^{(0)}\left)-\frac{1}{k}{\sum}_{k=1}^KP\left({h}_j=1\ \right|{v}^{(k)}\right)\\ {}P\left({h}_j^{(k)}=1\ \right|v\Big)= sigmoid\left({\sum}_{i=1}^n{W_{ij}}^{\ast }{v}_i+{c}_j\right)\\ {}P\left({v}_i^{(k)}=1\ \right|h\Big)= sigmoid\left({\sum}_{j=1}^m{W_{ij}}^{\ast }{h}_j+{b}_i\right)\end{array}\right. $$

where one step of Gibbs sampling (k = 1) is chosen to train the RBM in our method. More details of training RBM are described in [12].

Multiple RBMs stacked in our deep restricted Boltzmann machine are trained in a stepwise fashion, in which the hidden data of a trained RBM is fed as visible input data to next RBM. This process is repeated multiple times to transform the original input data into multiple non-linear representations denoted by hidden layers. A standard neural network with a linear regression output node is added at the top of the last hidden layer of multiple RBMs to predict torsion angles. The entire deep restricted Boltzmann machine consisting of the input layer, hidden layers, and the output layer is fine-tuned by traditional back-propagation of the errors between predicted output and true output to adjust the parameters, as described in the section "Deep neural network" of training a standard neural network.

#### Deep recurrent neural network

Recurrent neural network is one generalization of traditional feed-forward neural network, which is developed to handle sequential data. Recurrent neural network has achieved good performance on numerous bioinformatics problems, such as secondary structure prediction [20, 43]. Different from standard neural network that uses one sliding fixed-size window, recurrent neural network can recognize patterns in sequences of variable lengths. The sliding window approach can only learn the short-range dependency of residues within the window, and the inputs from different windows are independent of each other. Our deep recurrent neural network calculates the output at a specific position (or time) not only from all the inputs at the position (or a fixed-size window centered at the position), but also outputs of the previous position (or time), as shown in Fig. 3b. For a simple network with one hidden layer, the calculation can be described by the following equation:

7 $$ \left\{\begin{array}{c}\kern0.5em Input\kern0.75em Layer:\kern1.00em {Input}_i^{(t)}\kern0.75em i=1,2,\dots K,t=1,\dots, L\kern12.5em \\ {}\  Hidden Layer:\left\{\begin{array}{c}{I}_i^{(t)}={\sum}_j{W_{ij}}^{\ast }{Input}_j^{(t)}+{\sum}_k{U}_{ik}\ast {O}_k^{\left(t-1\right)}+\kern0.75em {b}_{h_i}^{(t)}\kern0.5em \\ {}{O}_i^{(t)}=\frac{1}{1+{e}^{-{I}_i^{(t)}}}\kern7.25em \\ {}i=1,2,\dots .K;j=1,2,\dots .M;\kern0.5em t=1,\dots .,L\end{array}\right.\\ {} Output Layer:\kern1.25em {Y}_i^{(t)}={\sum}_j{V_{ij}}^{\ast }{O}_j^{(t)}+{b}_{o_i}^{(t)}\kern0.5em \end{array}\right. $$

where the ${I}_i^{(t)}$, ${O}_i^{(t)}$ and ${b}_{h_i}^{(t)}$ is the input, output and bias of *i* ^*th*^ node for residue *t* in the first hidden layer, respectively, the ${Input}_i^{(t)}$ is the *i* ^*th*^ feature for residue *t*, *U*_*ik*_ is the weight connecting the output *j* in the hidden layer for residue *t-1* with the node *i* in the hidden layer for residue *t*. And the output in the output layer is calculated by a linear activation function as in Eq. 1. The weights of recurrent network can be tuned by back-propagation through time (BPTT) [44]. The SFO algorithm [36] was used to adjust the parameters (weights).

#### Deep recurrent restricted Boltzmann machines

Similar as traditional neural network, recurrent neural network may suffer the problem of vanishing gradient during training [45]. For example, a state-of-art method bidirectional recurrent neural network for protein secondary structure prediction can only capture long term dependency up to 15 amino acids from two directions [43]. Inspired by the pre-training method applied in deep belief network [39, 46] for mitigating the problem of vanishing gradient, we integrate the restricted Boltzmann machine with recurrent neural network to design a Deep Recurrent Restricted Boltzmann Machine for torsion angle prediction.

In DReRBM, the energy function at residue *t* is adjusted to include the output of hidden nodes at residue *t – 1*. The overall energy and probability model is described as the following equation:

8 $$ \left\{\begin{array}{c}E\left(v,h\right)=-\sum \limits_i\left({b}_i+\sum \limits_k{V_{ik}}^{\ast }\ {h}_k^{\left(t-1\right)}\right){v}_i-\sum \limits_j\left({c}_j+\sum \limits_k{U_{jk}}^{\ast }\ {h}_k^{\left(t-1\right)}\right){h}_j-\sum \limits_{i,j}{h}_j{v}_j{w}_{ij}\\ {}p\left(v,h\right)=\frac{e^{-E\left(v,h\right)}}{\sum \limits_{h,v}{e}^{-E\left(v,h\right)}}\\ {}p(v)=\sum \limits_h\frac{e^{-E\left(v,h\right)}}{\sum \limits_{h,v}{e}^{-E\left(v,h\right)}}\\ {}p(h)=\sum \limits_v\frac{e^{-E\left(v,h\right)}}{\sum \limits_{h,v}{e}^{-E\left(v,h\right)}}\end{array}\right. $$

where the *v*_*i*_ and *h*_*j*_ are the value of *i* ^*th*^ visible node and *j* ^*th*^ hidden node, *b*_*i*_ and *c*_*j*_ are the bias of *i* ^*th*^ visible node and *j* ^*th*^ hidden node, *w*_*ij*_ is the weight connecting the *i* ^*th*^ visible node and *j* ^*th*^ hidden node, *V*_*ik*_ is the weight connecting the *i* ^*th*^ visible node at time-stamp (*t*) with *k* ^*th*^ hidden node at time-stamp (*t-1*), and *U*_*jk*_ is the weight connecting the *j* ^*th*^ hidden node at time-stamp (*t*) with *k* ^*th*^ hidden node at time-stamp (*t-1*). In our architecture, each time-stamp represents a residue in a different position. In this energy function, we assume the dependency effects between two consecutive time-stamps is applied on the bias of both visible nodes and hidden nodes so that pre-training by RBM might better capture the correlation between inputs. The gradient of parameters can be calculated in Gibbs sampling as:

9 $$ \left\{\begin{array}{c}\frac{\partial lnP\left(\theta \right)}{\partial {W}_{ij}}=P\left({h}_j=1\ \right|{v}^{(0)}\left){}^{\ast }{v}_i^{(0)}-\frac{1}{k}{\sum}_{k=1}^KP\left({h}_j=1\ \right|{v}^{(k)}\right){}^{\ast }{v}_i^{(k)}\ \\ {}\frac{\partial lnP\left(\theta \right)}{\partial {b}_i}={v}_i^{(0)}-\frac{1}{k}{\sum}_{k=1}^K{v}_i^{(k)}\\ {}\frac{\partial lnP\left(\theta \right)}{\partial {c}_j}=P\left({h}_j=1\ \right|{v}^{(0)}\left)-\frac{1}{k}{\sum}_{k=1}^KP\left({h}_j=1\ \right|{v}^{(k)}\right)\\ {}\frac{\partial lnP\left(\theta \right)}{\partial {V}_{ik}}={h_k^{\left(t-1\right)}}^{\ast }\ \left[{v}_i^{(0)}-\frac{1}{k}{\sum}_{k=1}^K{v}_i^{(k)}\right]\\ {}\frac{\partial lnP\left(\theta \right)}{\partial {U}_{jk}}={h_k^{\left(t-1\right)}}^{\ast }\ \left[\ P\left({h}_j=1\ \right|{v}^{(0)}\right)-\frac{1}{k}{\sum}_{k=1}^KP\left({h}_j=1\ \right|{v}^{(k)}\left)\right]\\ {}P\left({h}_j^{(k)}=1\ \right|v\Big)= sigmoid\left({\sum}_{i=1}^n{W_{ij}}^{\ast }{v}_i+{c}_j\right)\\ {}P\left({v}_i^{(k)}=1\ \right|h\Big)= sigmoid\left({\sum}_{j=1}^m{W_{ij}}^{\ast }{h}_j+{b}_i\right)\end{array}\right.\kern7.75em $$

The training of RBM follows the same strategy described in [12], and the architecture is fine-tuned by algorithm of back-propagation through time and the SFO optimization method [36].

### Evaluation measure

We used Mean Absolute Error (MAE) to evaluate the prediction of phi and psi angles. The MAE is the average absolute difference between predicted angles (*P*) and experimentally determined angles (*E*) for all residues. Here, both *P* and *E* are in the range of [−180,180]. A direct subtraction of the two values may result in an artificial MAE >180. To rule out the artificial effect, we make a transformation of the predicted angles before comparing them as follows.

10 $$ \left\{\begin{array}{c}{P}^{\prime },\kern4.5em if\ \left|{P}^{\prime }-E\right|\le {180}^o\\ {}{P}^{\prime }+{360}^o,\kern1.25em if\ {P}^{\prime }-E\le -{180}^o\\ {}{P}^{\prime }-{360}^o,\kern1.25em if\ {P}^{\prime }-E\ge {180}^o\end{array}\right. $$

Where *P* ^′^ is the original value of the predicted torsion angles. Paired t-test are also applied to check the statistical significance between the performances of different methods.

## Results and discussions

We evaluated both normal deep learning models (DNN and DRBM) and deep recurrent learning models (DRNN and DReRBM). We also compared our methods with two other torsion angle prediction methods SPIDER2 and ANGLOR. In the following sections, firstly we assessed the impact of different feature combinations on the performance of DRBM. Then we identified the optimal window size for each of the four deep learning models and tested different memory sizes for two recurrent deep learning models (DRNN and DReRBM). Finally, we compared and analyzed the results of the six methods including our four in-house deep learning methods, SPIDER2, and ANGLOR.

### Impact of different feature combinations

We used 7 different features including two new features (predicted contact number and the error distribution of torsion angles predicted from sequence fragments) with our deep learning methods. Table 1 compares the performance of different feature combinations with DRBM on the test dataset. The DRBM was trained on the training data with window size of 17 and the architecture of three hidden layers of 500_200_50 (i.e. 500 nodes in the first hidden layer, 200 nodes in the second hidden layer, and 50 nodes in the third hidden layer). Among all the single features (Part 1 of Table 1), PSSM has the MAE of 23.28 and 35.12 for phi and psi angles, which has the best “avg” value. And our two new features performed better than the three common features (physicochemical, solvent accessibility and disorder). We tested two kinds of secondary structure features (3-state secondary structure prediction and 8-state secondary structure prediction) and two kinds of contacts number features (real-value contact number prediction and 15-class contact number prediction). The 8-state secondary structure feature achieved better performance than 3-state secondary structure, and the 15-class contact number probability prediction was more effective than the predicted real-value contacts number. To avoid redundancy in the features, we chose to use 8-class secondary structure feature and 15-class contact number probability feature with all our deep learning methods in this study.

**Table 1:** The Mean Absolute Error (MAE) of different feature combinations with the DBRM method

Number of features	Feature combination^a^	phi	psi	avg^b^
1	***PSSM***	**23.28**	**35.12**	**29.2**
	8-state secondary structure (8stateSS)	25.12	33.52	29.32
	Contacts_number_15_classes (CN15)	25.58	37.26	31.42
	Error_distribution_of_fragment_based_angles (fragsion)	24.24	40	32.12
	3-state secondary structure (3SS)	25.8	38.95	32.38
	Contacts_number_1_real_value (CN1)	26.92	44.71	35.82
	7 physicochemical properties (7PC)	27.27	52.18	39.73
	Solvent_accessibility (SA)	29.15	53.84	41.5
	Disorder	30.8	64.69	47.75
2	***PSSM_8stateSS***	**22.18**	**30.73**	**26.46**
	PSSM_CN15	22.41	33.14	27.78
	PSSM_Fragsion	22.19	34.29	28.24
	PSSM_7PC	22.42	35.75	29.09
	PSSM_DISORDER	22.96	35.23	29.1
	PSSM_SA	23.47	35.53	29.5
3	***PSSM_8stateSS_7PC***	**21.48**	**30.36**	**25.92**
	PSSM_8stateSS_Fragsion	21.63	30.72	26.18
	PSSM_8stateSS_CN15	21.99	30.12	26.06
	PSSM_SS8_Disorder	22.91	31.08	27
	PSSM_8stateSS_SA	23.09	31.41	27.25
4	***PSSM_8stateSS_7PC_CN15***	**21.48**	**30.27**	**25.88**
	PSSM_8stateSS_7PC_SA	21.88	30.89	26.39
	PSSM_8stateSS_7PC_Disorder	22.17	30.97	26.57
	PSSM_8stateSS_7PC_Fragsion	22.08	31.11	26.595
5	***PSSM_8stateSS_7PC_CN15_Disorder***	**21.54**	**29.94**	**25.74**
	PSSM_8stateSS_7PC_CN15_SA	21.93	30.39	26.16
	PSSM_8stateSS_7PC_CN15_Fragsion	21.81	30.83	26.32
6	***PSSM_8stateSS_7PC_CN15_Disorder_Fragsion***	**21.11**	**30.33**	**25.72**
	PSSM_8stateSS_7PC_CN15_Disorder_SA	22.24	30.60	26.42
7	***PSSM_8stateSS_7PC_CN15_Disorder_Fragsion_SA***	**21.36**	**29.83**	**25.6**

^a^Features combination: for example “PSSM_8stateSS” represent the combination of PSSM and 8-state secondary structure as input features. The bold font denotes the best combination selected for a specific number of features in terms of the average MAE of phi and psi angles

^b^avg.: Average of phi and psi values for each features combination

Part 2 of Table 1 shows the results of combining PSSM with every other feature. Except for solvent accessibility, every other feature combination improved the prediction accuracy than using PSSM alone, suggesting that directly adding each of five other features on top of PSSM is beneficial. For instance, combining PSSM with the error distribution of fragment-based angles has MAE of 22.19 and 34.29 for phi and psi angles, and combining predicted contacts number with PSSM has MAE of 22.41 and 33.14 for phi and psi angles, which is better than MAE of 23.28 and 35.12 of using PSSM alone.

We continued to add one additional feature into the best set of feature of the previous round progressively to find good combination of 3 features, 4 features, 5 features, and all the 7 features (see Parts 3–7 of Table 1). We found that this forward feature selection can give us very good or even best feature combinations for a specific feature number. In view of the whole results from the Table 1, we found that, if every time we choose the best feature combination as basis to combine more features, most of the time we can get better result in the next step. The best combination for each feature number tends to include either contact number feature or the error distribution of fragment-based angles, indicating that the two new features can improve the prediction accuracy.

We consider PSSM, solvent accessibility, secondary structure, protein disorder and 7 physicochemical properties as five standard features. In order to evaluate the performance improvement induced by adding the two novel features, we performed the experiments with different features sets with or without either one or both of the two novel features as follows: standard features (Feature set 1), standard features plus contacts number (Feature set 2), standard features plus fragsion (Feature set 3), and standard features plus contacts number and fragsion (Feature set 4). These experiments was conducted using the DRBM model. Table 2 shows that including either contact number or fragsion can slightly improve the prediction of phi and psi angle, while including both features can further improve the prediction accuracy, especially for the psi angle, whose prediction accuracy is improved by 10.1% if two novel features are added.

**Table 2:** The prediction performance of using the standard features with two novel features

	Feature set 1	Feature set 2	Feature set 3	Feature set 4
phi	22.16	21.85	22.05	**21.36**
psi	33.21	32.81	32.76	**29.83**

Note:

Feature set 1: standard features

Feature set 2: standard features plus contact number

Feature set 3: standard features plus fragsion feature

Feature set 4: standard features plus contact number and fragsion

### Effect of different window sizes

A sliding window approach was used often to extract the local sequence profile for each residue in a sequence, which was used as input to our four deep learning methods. We tested window size ranging from 1 to 17 with our four deep learning architectures having 3 hidden layers consisting of 500, 200 and 50 nodes respectively.

Table 3 reports the accuracy of phi and psi angle predictions of four different methods with different window size. It is shown that the accuracy increases as the window size increases at the beginning, reaches the highest at a certain size, and then starts to decrease as the size continues to increase. This is because increasing window size at the beginning may incorporate more information than noise, leading to better performance, but after a specific threshold, increasing window size may include more remote information that contains more noise than signal, leading to worse performance. According to Table 3, the best local window size for DRBM is 7, which has a MAE of 20.84 and 28.85 for phi and psi angles respectively. Similarly, the best window size for DRNN is also 7. The best window size for DReRBM is 3. For DNN, the best window size is 11, which has a MAE of 21.04 and 29.06 for phi and psi angles. Compared with normal deep networks, deep recurrent networks can work well with smaller window sizes because they can use the output information from previous positions as input recursively. Larger window size generally performs better than window size equals to “1” suggests that local context information is important for torsion angle prediction.

**Table 3:** The prediction performance of phi and psi angles of using different local window sizes with four deep learning methods

Torsion Angle	Window size^a^	Features^b^	DRNN	DReRBM	DNN	DRBM
phi	1	56	21.09	21.81	22.13	21.95
	3	*168*	20.52	**20.77**	21.49	21.07
	5	280	20.39	20.92	21.24	20.89
	7	392	**20.39**	21.03	21.22	**20.84**
	9	504	20.40	20.95	21.28	21.62
	11	616	20.49	20.88	**21.04**	21.57
	13	728	20.56	20.98	21.27	21.79
	15	840	20.63	21.12	21.19	21.69
	17	952	20.69	21.04	21.38	21.66
psi	1	56	31.68	32.93	33.55	33.02
	3	*168*	29.29	**29.86**	30.14	29.74
	5	280	28.96	29.94	29.25	28.92
	7	392	**28.85**	30.11	29.25	**28.85**
	9	504	28.86	29.94	29.38	29.61
	11	616	29.06	29.95	**29.06**	29.75
	13	728	29.27	30.13	29.38	30.19
	15	840	29.44	30.48	29.24	30.25
	17	952	29.72	30.36	29.54	30.33

^a^Number of window size range from 1 to 17

^b^Number of features as input for the deep learning model. For each residue, we used 7 kinds of features, represented by 56 numbers

The bold fond denotes the best result for each method

### Effect of different memory lengths on deep recurrent networks

Different with traditional deep networks, deep recurrent networks assume that the output of current position (or time) depends on that of the previous positions. Therefore, deep recurrent networks have a “memory”, which captures information about what has been calculated so far. In theory, recurrent networks can make use of the information from a long previous sequence, but in practice they are mostly limited to looking back in a few steps due to vanishing gradients during back-propagation or decreasing signal to noise ratio. In this work, we tested DRNN and DReRBM on five different memory lengths (i.e. 5,10,15,20,25) and the results are shown in Table 4. For DRNN, smaller memory lengths (5, 10, 15) yield better performance than larger memory lengths (20, 25), but DReRBM obtained comparable results use different memory lengths. This indicates that DReRBM can use longer memory length than DRNN. In this study, since smaller memory lengths perform similarly or better, we chose to use the memory length of 5 to train both DRNN and DReRBM. Compared to the traditional deep feed-forward networks that make predictions based only on the information in a fixed-size local window, DRNN and DReRBM predict torsion angles using the information from the entire input sequence by propagating information through recurrent networks, which leads to the improved performance of the recurrent methods (DRNN and DReRBM) over the deep feed-forward network based methods (DNN and DRBM).

**Table 4:** The prediction performance of phi and psi angles of using different memory lengths for DRNN and DReRBM

Torsion Angle	Memory Length	DRNN		DReRBM	
phi	5	**20.52**	**0.595**	**20.77**	**0.583**
	*10*	20.53	0.600	20.95	0.581
	15	20.74	0.589	20.81	0.582
	*20*	22.20	0.539	20.90	0.580
	25	22.16	0.541	20.82	0.586
psi	5	**29.29**	**0.699**	**29.86**	**0.695**
	*10*	29.35	0.700	30.02	0.694
	15	29.74	0.696	29.94	0.694
	20	32.82	0.670	30.02	0.696
	25	32.78	0.671	29.89	0.698

### Comparison of different methods on independent test data

We performed 5-fold cross validation of our four methods on the training data set and chose the appropriate features combination, window size, and/or memory lengths for each of our deep learning method. For the non-recurrent models DNN and DRBM, we assessed the effect of different numbers of hidden layers. As shown in Table 5, three hidden layers can achieve similar performance as 5 hidden layers, which is better than other numbers of hidden layers. Therefore, we finally used a simpler three-hidden-layer architecture (500_200_50) consisting of 500, 200 and 50 nodes for each hidden layer, respectively. After these methods with selected parameters were trained on the training dataset, they were blindly tested on the test dataset consisting of 232 proteins.

**Table 5:** The effect of the number of hidden layers on DRBM and DNN

Hidden layers	DRBM		DNN	
	Phi (MAE)	Psi (MAE)	Phi (MAE)	Psi (MAE)
2 layers	21.76	30.57	21.33	31.02
3 layers	21.13	29.43	21.21	30.05
4 layers	21.77	31.02	21.65	30.88
5 layers	21.23	29.47	21.25	29.97

Table 6 reports the MAE on the test data for DNN, DRBM, DRNN, DReRBM, SPIDER2 and ANGLOR. DReRBM has the lowest MAE of 20.49 and 29.06 for phi and psi angles, which is better than 20.88 and 31.64 of SPIDER2 and much better than 24.72 and 44.42 of ANGLOR. Overall, our four methods achieved the performance of phi angle prediction that was comparable to a state-of-the-art method SPIDER2 and made notable improvements on the prediction of psi angles. Our experiment also shows that the two deep recurrent networks (DRNN and DReRBM) performed better than the two standard deep networks (DNN and DRBM).

**Table 6:** The prediction performance of torsion angles on six different methods

DRNN		DReRBM		DNN		DRBM		SPIDER2		ANGLOR	
phi	Psi	phi	psi	phi	psi	phi	Psi	phi	psi	phi	psi
20.78	29.85	**20.49**	**29.06**	21.14	29.35	20.75	29.07	20.88	31.64	24.72	44.42

### Comparison of our methods with SPIDER2 on CASP 12 targets

Table 7 shows the accuracy of our four deep learning models and SPIDER2 on 11 CASP12 free modeling targets. For the phi angle, the MAE of four deep learning methods and SPIDER2 are comparable to each other. For the psi angle prediction, the recurrent methods produce lower MAE than the other methods. Compared to SPIDER2, DReRBM can achieve 4.4% improvement on MAE of the psi angle. The *p*-values of paired t-test between each pair of methods are shown in Table 8. For each method, the MAE value for each residue on phi and psi angles were calculated, and paired t-test were applied to the results of different methods. Table 8 shows that the DReRBM is significantly more accurate than SPIDER2 on both phi and psi angle. Especially, the more significant improvement on psi angle is achieved by DReRBM, which is consistent with the results in Table 6. In terms of the running time on the CASP12 dataset, SPIDER2 takes about 37 s and our methods take about 863 s to make prediction on average. The relatively longer running time for our method is because of the time needed by third-party tools to generate input features. Once the features are generated, our methods can make predictions in seconds.

**Table 7:** The prediction performance of torsion angles on CASP12 free modeling targets

	DNN	DRBM	DRNN	DReRBM	SPIDER2
Phi (MAE)	22.68	22.72	22.38	22.47	22.61
Psi (MAE)	38.37	37.56	37.54	35.99	37.67

**Table 8:** The statistical significance (*p*-value) of the performance difference between our methods and SPIDER2

Method	DRBM		DRNN		DReRBM		SPIDER2	
	phi	psi	phi	psi	phi	psi	phi	psi
DNN	8.5E-02	1.2E-05	5.1E-03	5.8E-05	9.3E-03	2.6E-08	8.2E-02	2.0E-03
DRBM			8.4E-03	3.8E-01	3.3E-04	1.4E-07	1.4E-03	6.3E-04
DRNN					3.5E-02	5.5E-05	6.4E-03	8.9E-02
DReRBM							1.5E-03	1.5E-06

## Conclusions

In this study, we developed four different deep learning methods for protein torsion angle prediction. We tested various feature combinations, window sizes, memory lengths, and numbers of hidden nodes to study their impact on the prediction accuracy. Our experiment shows that the two new features (predicted contact number and error distribution of fragment-based torsion angles) are useful for torsion angle prediction, and recurrent deep learning architectures perform better than feed-forward deep learning architectures. Finally, we demonstrated that deep learning methods achieved the performance better than or comparable to the state of the art methods for torsion angle prediction on both independent datasets and CASP12 targets.

## Additional file

Additional file 1: Table S1. The 11 free-modeling targets in the most recent CASP12 (DOCX 12 kb)

## Abbreviations

BPTT: Back-Propagation Through Time
CD: Contrastive Divergence
DNN: Deep Neural Network
DRBM: Deep Recurrent Boltzmann Machine
DReRBM: Deep Recurrent Restricted Boltzmann Machine
DRNN: Deep Recurrent Neural Network
MAE: Mean Absolute Error
PDB: Protein Data Bank
PSSM: Position Specific Scoring Matrices
RBM: Restricted Boltzmann Machines
SFO: Sum-of-Functions-Optimizer
SVM: Support Vector Machines
