# Supplementary material for: Deep learning methods for protein torsion angle prediction
Source: BMC Bioinformatics. 2017 Sep 18;18:417. doi: 10.1186/s12859-017-1834-2 (PMC5604354; doi:10.1186/s12859-017-1834-2)
Supplement: Additional file 1: Table S1. — The 11 free-modeling targets in the most recent CASP12 (DOCX 12 kb) [file 12859_2017_1834_MOESM1_ESM.docx]

**Table S1. The 11 free-modeling targets in the most recent CASP12**

| Target | length |
| --- | --- |
| T0859 | 113 |
| T0862 | 93 |
| T0863 | 582 |
| T0864 | 246 |
| T0866 | 115 |
| T0869 | 104 |
| T0870 | 123 |
| T0886 | 229 |
| T0900 | 102 |
| T0904 | 311 |
| T0912 | 599 |
